# Bioinformatics Analysis and Experimental Validation of Key Genes Associated With Hypoxia and Ischemia in Myocardial Infarction

**DOI:** 10.1002/mgg3.70284

**Published:** 2026-09-07

**Authors:** Longsheng Zhang, Ning Liang

**Affiliations:** ^1^ Ward 1, Department of Tuberculosis HIV/AIDS Clinical Treatment Center of Guangxi (Nanning) and The Fourth People's Hospital of Nanning Nanning Guangxi China; ^2^ The First Affiliated Hospital of Guangxi Medical University Nanning Guangxi China; ^3^ Ward 1, Department of Cardiovascular Medicine The Third People's Hospital of Nanning Nanning Guangxi China

**Keywords:** hypoxia‐ischemia, myocardial infarction, protein–protein interaction, qPCR, random forest

## Abstract

**Background:**

This study aimed to screen and identify core hypoxia‐ischemia‐related genes associated with myocardial infarction (MI).

**Method:**

Two transcriptomic datasets, GSE97320 and GSE48060, were retrieved from the Gene Expression Omnibus (GEO) database. After data integration and batch effect elimination, differential expression analysis was performed to screen differentially expressed genes (DEGs), and the corresponding visualization analysis was conducted. Hypoxia‐ischemia‐related genes were acquired from the GeneCards database; hypoxia‐ischemia related genes (HIRGs) were subsequently identified by intersecting the retrieved genes with screened DEGs. Gene Ontology (GO) functional enrichment and Kyoto Encyclopedia of Genes and Genomes (KEGG) pathway enrichment analyses were implemented to explore the biological functions and underlying signaling pathways of HIRGs. A combination of protein–protein interaction (PPI) network analysis and random forest (RF) algorithm was applied to screen hub genes from HIRGs. The external GEO dataset GSE66360 was utilized to validate the expression patterns of candidate hub genes. Furthermore, an acute myocardial infarction (AMI) mouse model was established, and quantitative real‐time polymerase chain reaction (qPCR) was performed to detect the mRNA expression levels of hub genes in myocardial tissues for in vivo validation.

**Results:**

A total of 633 DEGs and 308 hypoxia‐ischemia‐related genes were screened in the present study, among which 21 overlapping HIRGs were obtained. PLAUR and IL1B were finally identified as two hub genes from HIRGs based on PPI network and random forest algorithm. The qPCR results revealed that the expression levels of PLAUR and IL1B were significantly upregulated in the AMI group compared with the sham operation group (*p* < 0.05).

**Conclusion:**

The present findings demonstrated that PLAUR and IL1B serve as pivotal genes involved in the pathological hypoxia‐ischemia process of AMI. These two genes may act as novel biomarkers and promising therapeutic targets for the recognition and clinical intervention of hypoxia‐ischemia injury following AMI.

## Graphical abstract

This study screened hypoxia‐ischemia hub genes for AMI through transcriptomic bioinformatics and animal tests. Merging two GEO datasets yielded 633 DEGs; intersection with GeneCards hypoxia genes produced 21 HIRGs. PPI network and random forest identified core PLAUR and IL1B, whose diagnostic value was validated by GSE66360 via ROC, calibration, and DCA curves. Mouse AMI model qPCR confirmed their myocardial upregulation, marking them as AMI hypoxic injury biomarkers and therapeutic targets.

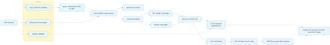

## 1 Introduction

Myocardial infarction ranks among the leading causes of cardiovascular disease mortality worldwide (Chen et al. 2025). Its core pathophysiological mechanism involves acute coronary artery occlusion inducing myocardial tissue hypoxia and ischemia, subsequently leading to extensive myocardial cell necrosis, cardiac remodeling, and irreversible cardiac dysfunction (Schumacher and Kramann 2023). Although clinical interventions such as percutaneous coronary intervention (PCI) and anticoagulation have significantly improved short‐term patient outcomes (Reddy et al. 2024), long‐term complications like ischemia–reperfusion injury and chronic heart failure continue to severely impact patients' quality of life. Deepening our understanding of the molecular regulatory mechanisms governing cardiomyocytes under hypoxic–ischemic conditions is crucial for identifying novel therapeutic targets for MI. As the initiating factor in MI progression, ischemia‐hypoxia rapidly activates multiple intracellular signaling pathways within cardiomyocytes. By regulating the expression of key downstream genes, it mediates the balance between cell survival and death (De Caterina and Brown 2021). Therefore, systematically screening and deciphering critical genes within the ischemic–hypoxic microenvironment of MI not only deepens our understanding of MI pathophysiology but also provides novel theoretical foundations and experimental bases for developing targeted gene therapies and enhancing myocardial protection strategies.

## 2 Materials and Methods

### 2.1 Data Download and Preprocessing

**FIGURE 1:**
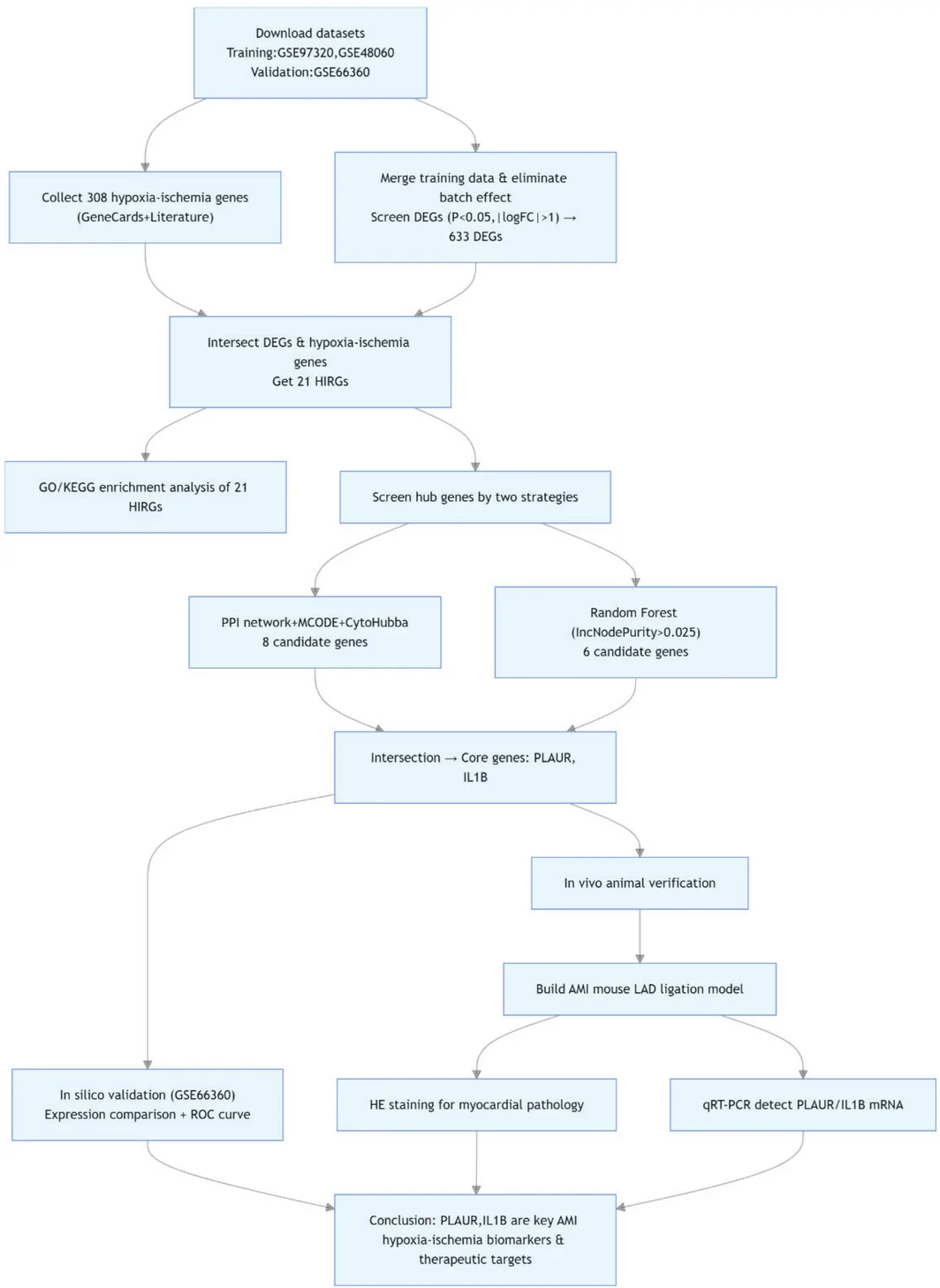
Flowchart of the analysis of this study.

Figure 1 shows the flowchart of this study. The technical workflow of this study is illustrated in Figure 1. Using “myocardial infarction” as the search term, three datasets (GSE48060, GSE97320, and GSE66360) were screened and downloaded from the GEO database. All datasets were generated based on the GPL570 platform (HG‐U133_Plus_2) Affymetrix Human Genome U133 Plus 2.0 Array, and peripheral whole blood samples included in the above three datasets were collected from AMI patients and healthy individuals (Dai et al. 2024; Wang et al. 2022; Zhou et al. 2025).

Genes associated with hypoxia‐ischemia were retrieved from the GeneCards database with “hypoxia” as the keyword (Fishilevich et al. 2017). Protein‐coding regulatory genes with a relevance score greater than 5 were reserved, and a total of 123 hypoxia‐ischemia‐related genes were obtained. Additionally, 228 hypoxia‐associated genes were summarized from previously published relevant literatures (Fu et al. 2021; Mo et al. 2020; Zhang et al. 2020). After integrating the two sets of genetic data and removing duplicate genes, a final collection of 308 hypoxia‐related genes was acquired for subsequent analysis.

All data utilized in the present study were acquired from publicly accessible databases; therefore, ethical approval from the institutional review board was not required.

### 2.2 HIRGs Screening

GSE48060 and GSE97320 datasets were applied for the screening of DEGs. The two datasets were merged, and the integrated data was further processed to eliminate batch effects. The ComBat function embedded in the R package “sva” (R version 4.4.1) was utilized to mitigate cross‐dataset batch bias. Subsequently, the “limma” package was employed to conduct differential expression analysis. Genes with a threshold of *p* < 0.05 and |logFC| > 1 were identified as DEGs between the AMI group and the control group. The “pheatmap” package and “ggplot2” package were separately adopted to generate clustering heatmaps and volcano plots. Finally, hypoxia‐ischemia‐related genes were intersected with the screened DEGs to obtain HIRGs.

### 2.3 GO and KEGG Analysis

Functional annotation of GO and KEGG pathway enrichment analyses was performed on HIRGs using the R package clusterProfiler. Adjusted *p‐*value (P. adj) less than 0.05 was set as the cutoff criterion. The enrichment analysis outcomes were visualized via the ggplot2 R package (Yu et al. 2012).

### 2.4 Construction and Modular Analysis of Protein–Protein Interaction (PPI) Network

To systematically characterize the interrelationships among candidate proteins, a PPI network was constructed via the STRING database (Version 11.5; http://string‐db.org) (Franceschini et al. 2012). This online analytical platform is capable of capturing multiple interaction patterns among proteins, including direct physical binding and upstream/downstream regulatory correlations. Only paired interactions with a combined confidence score over 0.4 were retained for subsequent analyses. The finalized PPI network was imported into Cytoscape software (Version 3.9.0; http://www.cytoscape.org) (Smoot et al. 2011) for intuitive graphical visualization. The molecular complex detection (MCODE) plugin embedded in Cytoscape was utilized to partition functional modules within the network, with the parameters configured as follows: K‐core value of 2, degree cutoff set to 2, maximum depth limited to 100, and node score cutoff set at 0.2.

### 2.5 Identification and Bioinformatics Analysis of Hub Genes

Hub genes underlying the hypoxia‐ischemia regulatory network were screened using the cytoHubba plugin of Cytoscape. Six mainstream computational algorithms, encompassing MCC, MNC, Degree, Closeness, Radiality, and EPC, were adopted to comprehensively evaluate gene connectivity and centrality for the identification of robust hub candidates. Furthermore, the co‐expression regulatory network of screened hub genes was established using GeneMANIA (http://www.genemania.org) (Warde‐Farley et al. 2010). As a powerful and reliable bioinformatics tool, GeneMANIA integrates multi‐omics data covering genomics and proteomics profiles. It can not only elucidate intrinsic correlations among input genes but also expand the gene set by supplementing additional genes with analogous biological functions.

### 2.6 Random Forest (RF) Algorithm for Screening Hub HIRGs

Apart from network‐based analysis, the RF ensemble learning model was implemented to screen signature genes from the overall HIRG pool. As a sophisticated ensemble learning technique, the RF model generates numerous independent decision trees throughout the training phase and synthesizes the outputs of all decision trees to accomplish classification and regression tasks. Gene importance rankings were determined by quantifying the individual contribution of each gene toward enhancing the overall classification performance of the model. In this study, the RF model was configured with 500 decision trees for feature recognition, and the optimal model structure was determined by selecting the tree number corresponding to the minimum error rate. Gini impurity reduction was adopted to prioritize candidate genes based on feature importance; genes with an importance score greater than 0.025 were regarded as pivotal candidates. Ultimately, the overlapping genes shared by RF algorithm prediction and PPI network analysis were extracted and defined as the core HIRGs for subsequent research.

### 2.7 Evaluation of Diagnostic Performance for Core Genes

The GSE66360 dataset was utilized as an external validation cohort to verify the differential expression patterns and diagnostic efficacy of identified core genes. The R‐based “ggpubr” package was employed to generate box plots for visualizing the expression profiles of hub genes across both training and validation datasets, thereby facilitating intergroup comparison between AMI and normal samples. To further evaluate the diagnostic potency of candidate biomarkers for AMI, receiver operating characteristic (ROC) curves were plotted, and the area under the curve (AUC) was calculated to quantify the capability of targeted genes in distinguishing AMI specimens from healthy controls. Logistic regression was used to establish the binary predictive model with *PLAUR* and IL1B as predictive variables for AMI status. Based on this logistic model, calibration curves were plotted via the R “rms” package to assess the agreement between model‐predicted probabilities and actual observed outcomes. Decision curve analysis (DCA) was implemented using the R “rmda” package to calculate net clinical benefit at varying threshold probabilities and compare the performance of single‐gene and combined two‐gene prediction models.

### 2.8 Animal Models

G*Power 3.1 was utilized to calculate the sample size for animal studies based on an independent samples *t*‐test. Calculation parameters were set to an effect size of 1.2, statistical power of 0.8, and a significance level of 0.05. The computational results demonstrated that eight animals per group was the minimal quantity required. To accommodate potential surgical complications and low modeling efficiency associated with acute myocardial infarction surgery, 10 mice were assigned to both the sham operation group and MI model group.

Healthy 8‐week‐old male C57BL/6J mice with body weights ranging from 20 g to 25 g were purchased from Guangdong Weitong Lihua Laboratory Animal Technology Co. Ltd. (SCXK (Yue)‐2022–0063). All experimental rodents were housed in a dedicated SPF‐level facility at Guangxi Medical University, where the ambient temperature was maintained at 20°C–25°C and relative humidity stabilized at 50%–60%. Autoclaved diet and purified water were continuously supplied to satisfy daily growth demands. All in vivo procedures were implemented in accordance with the 8th version of internationally recognized laboratory animal care guidelines, and the entire experimental scheme obtained formal approval from the Animal Ethics Committee of The First Affiliated Hospital of Guangxi Medical University (Approval ID: 2025‐D0343).

Animals were equally categorized into two subgroups using a random sampling method. Briefly, mice received intraperitoneal anesthesia with 1.2% tribromoethanol at a dosage of 0.02 mL/g, followed by operative area sterilization using 75% alcohol. With Lead‐II ECG signals dynamically monitored and mechanical ventilation well configured, a left parasternal incision was created to expose the myocardium, and the mid‐segment of the left anterior descending coronary artery was ligated to establish an acute ischemic model (Bo et al. 2024).

Multiple objective indicators covering ECG alterations and histological manifestations were adopted to differentiate valid and invalid models. After complete layered suturing, experimental subjects regained spontaneous respiration and recovered on a thermostatic heating platform before rehousing. Postoperative adverse events were closely tracked throughout the whole period, while sham‐operated animals underwent identical surgical exposure without coronary ligation.

After 24 h of sustained myocardial ischemia (Jiang et al. 2022; Zhu et al. 2024), eligible mice were humanely euthanized via excessive anesthetic injection. Harvested cardiac tissues were separated into two portions, which were prepared for routine HE histological staining and qPCR quantification, respectively; specimens from unqualified models were excluded from further statistical analysis.

### 2.9 HE Staining for Myocardial Tissue Assessment

Myocardial histological alterations were assessed via routine hematoxylin and eosin (HE) staining. Ischemic cardiac tissues were harvested, fixed, paraffin‐embedded, and cut into continuous sections with a standardized thickness of 4 μm. The obtained slices were sequentially processed through dewaxing, gradient rehydration, HE staining, and neutral gum sealing. Microscopic photographs were acquired under an optical microscope at 400× magnification for subsequent histological observation.

### 2.10 qPCR

Cardiac tissues were homogenized with 1 mL Trizol reagent and grinding beads for 1 min. The homogenates were centrifuged at 12,000 rpm for 5 min at 4°C. The collected supernatant was mixed with 200 μL chloroform and vortexed intensely. After 5 min of static placement, the mixture was re‐centrifuged at 12,000 rpm for 15 min at 4°C. The aqueous phase was transferred to a new tube, supplemented with 500 μL isopropanol for RNA precipitation, and incubated for 10 min.

Samples were centrifuged at 12,000 rpm for 10 min at 4°C to obtain RNA pellets. After supernatant removal, the pellets were washed with pre‐prepared 75% ethanol and centrifuged at 7500 rpm for 5 min at 4°C. The residual ethanol was carefully discarded, and RNA pellets were air‐dried at room temperature before being dissolved in 10 μL nuclease‐free water. RNA purity was determined using a NanoDrop 200 spectrophotometer, and samples with an OD260/OD280 ratio of 1.8–2.0 were used for subsequent experiments.

Purified total RNA was reverse‐transcribed into cDNA following Takara's official instructions. qPCR analysis was carried out to detect target gene expression (Firoozi et al. 2024). GAPDH was selected as the internal reference for data normalization, and all primer sequences were presented in Table 1.

**TABLE 1:** Primers used for qPCR.

Gene	Forward primer	Reverse primer
PLAUR	CTGTTGCTGGCGACTACCTG	TAACTCATGGTCCTGTTGGTCTTTT
IL1B	GCATCCAGCTTCAAATCTCGC	TGTTCATCTCGGAGCCTGTAGTG
GAPDH	CCTCGTCCCGTAGACAAAATG	TGAGGTCAATGAAGGGGTCGT

### 2.11 Statistical Methods

For measurement indicators conforming to gaussian distribution, they were described using mean ± standard deviation (x ± s) and contrasted using independent samples *t*‐tests. If measurement data did not fit normal distribution, described with median (IQR) and compared using rank‐sum tests. Statistical significance was defined as a *p*‐value < 0.05. All analyses were conducted using SPSS 23.0 statistical software, with graphical representations generated using GraphPad Prism 10.0.

## 3 Results

### 3.1 Identification of DEGs and HIRGs

**FIGURE 2:**
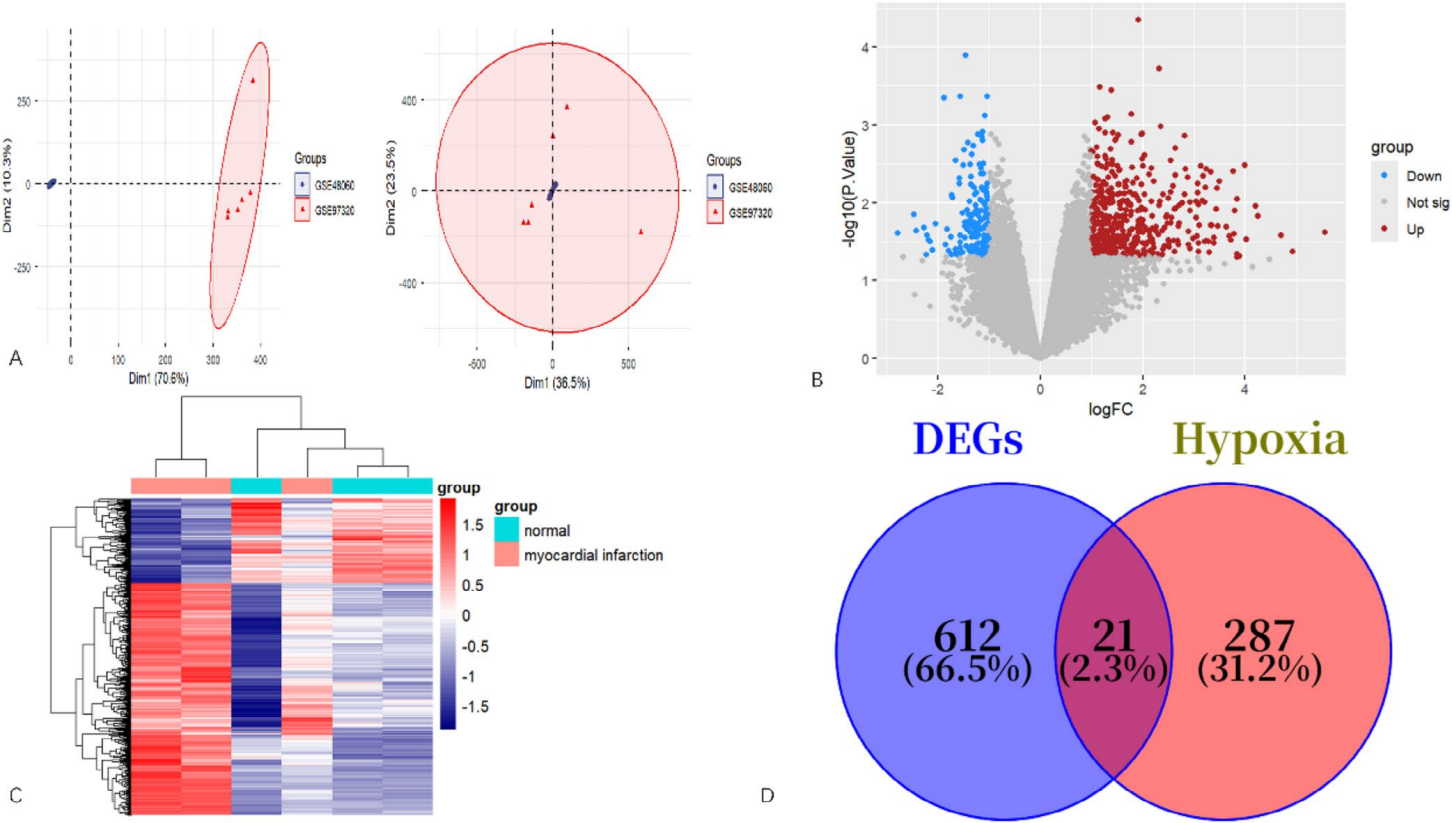
Screening and preliminary analysis of DEGs. (A) PCA of gene expression in AMI and normal samples. (B) Volcano plot of DEGs between two groups. (C) Expression heatmap of DEGs across all samples. (D) Venn diagram of overlaps between DEGs and hypoxia‐ischemia related genes.

The integrated dataset merged from GSE97320 and GSE48060 was adopted as the training cohort for differential gene screening. After data integration and batch effect elimination, principal component analysis (PCA) was performed. The results showed tight intra‐group aggregation and clear inter‐group separation between AMI and normal control samples, confirming reliable sample grouping and distinct transcriptomic characteristics (Figure 2A). A total of 633 DEGs were distinguished, consisting of 464 upregulated transcripts and 169 downregulated transcripts. Volcano plots and clustering heatmaps were generated to intuitively display the divergent expression profiles of these genes (Figure 2B,C). After overlapping the 633 DEGs with a pool of 308 hypoxia‐ischemia‐associated genes, 21 intersecting HIRGs were captured in total (Figure 2D). Full detailed information of these 21 HIRGs is summarized in Table 2.

**TABLE 2:** 21 HIRGs.

Gene symbol	LogFC	*p*	Level
NFIL3	2.320764	0.000189	Up
PLAUR	1.125209	0.002565	Up
ADM	2.171676	0.003618	Up
RRAGD	1.193983	0.006756	Up
PNRC1	1.325514	0.007311	Up
IL1B	2.031792	0.00779	Up
PTEN	1.562469	0.010609	Up
MMP9	2.621395	0.01203	Up
EGLN1	1.517706	0.018667	Up
TLR4	1.014454	0.018934	Up
SIAH2	1.938345	0.019124	Up
PPP1R15A	1.239328	0.024359	Up
CXCR4	1.735453	0.025989	Up
EPOR	1.019535	0.02832	Up
TGM2	1.8195	0.03679	Up
MXI1	2.617788	0.042784	Up
PTGS2	1.844896	0.04362	Up
PFKL	1.087875	0.044183	Up
CXCL8	1.87811	0.047021	Up
CYB5R3	1.596352	0.048469	Up
SELENBP1	3.894157	0.048504	Up

### 3.2 Functional Enrichment Analysis

**FIGURE 3:**
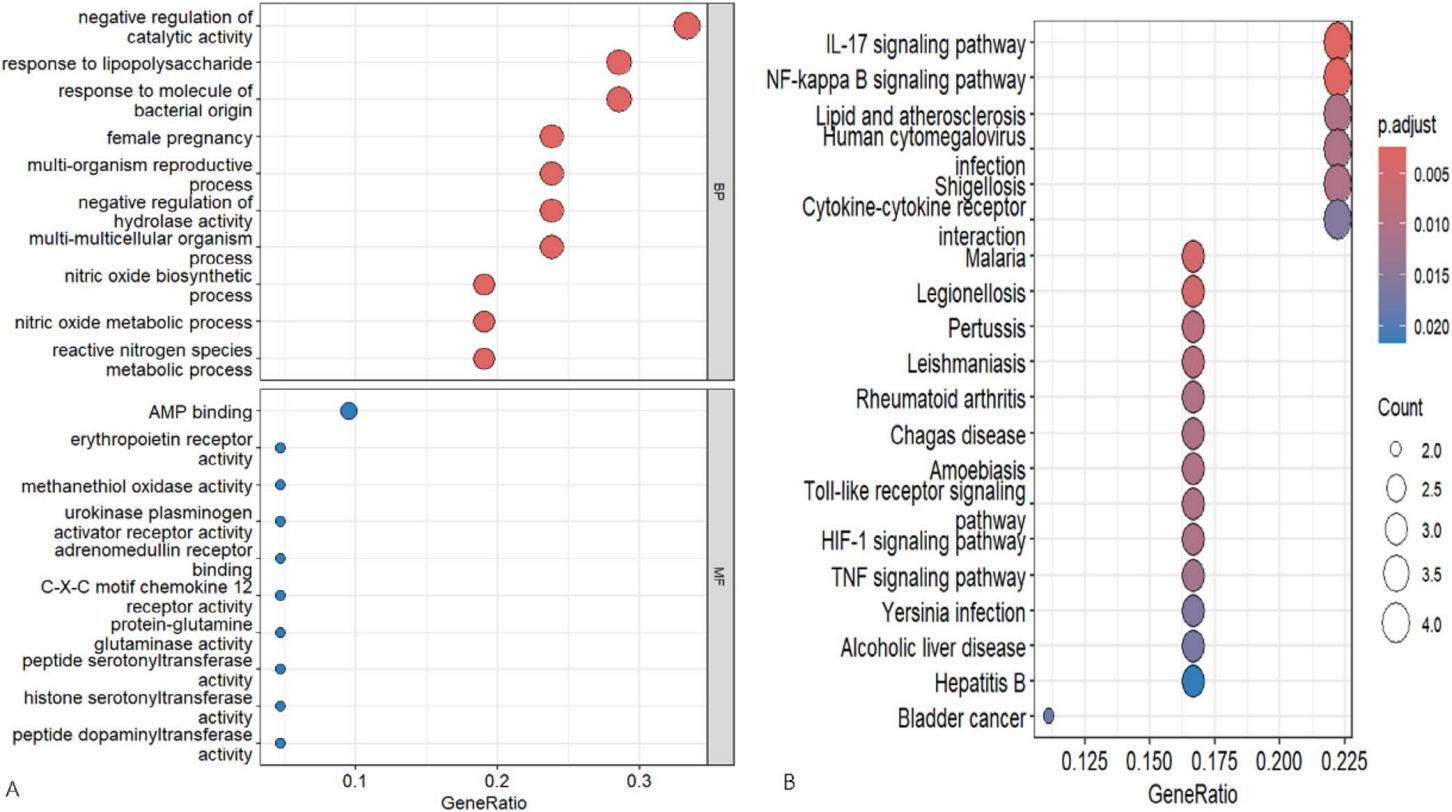
Enrichment Analysis (A) GO analysis bubble plot of 21 HIRGs. (B) KEGG pathway enrichment plot of the 21 HIRGs.

Enrichment analyses of GO and KEGG pathways were performed on the 21 identified HIRGs. Biological process (BP) subsets of GO enrichment revealed prominent enrichment terms including negative modulation of catalytic function, cellular responses toward lipopolysaccharide, reactions to bacterial‐derived molecules, and processes relevant to female gestation. Molecular function (MF) enrichment demonstrated predominant functional annotations such as adenosine monophosphate binding, erythropoietin receptor function, methanethiol oxidase catalytic capacity, as well as urokinase plasminogen activator receptor activity (Figure 3A).

KEGG pathway enrichment uncovered markedly enriched signaling cascades, namely the IL‐17 signaling axis, NF‐κB signal transduction pathway, lipid metabolism coupled with atherosclerotic progression, human cytomegalovirus infectious pathways, shigellosis infection routes, and cytokine‐cytokine receptor interaction networks (Figure 3B).

### 3.3 Screening of Core Genes

#### 3.3.1 Construction of PPI Network and Module Analysis

**FIGURE 4:**
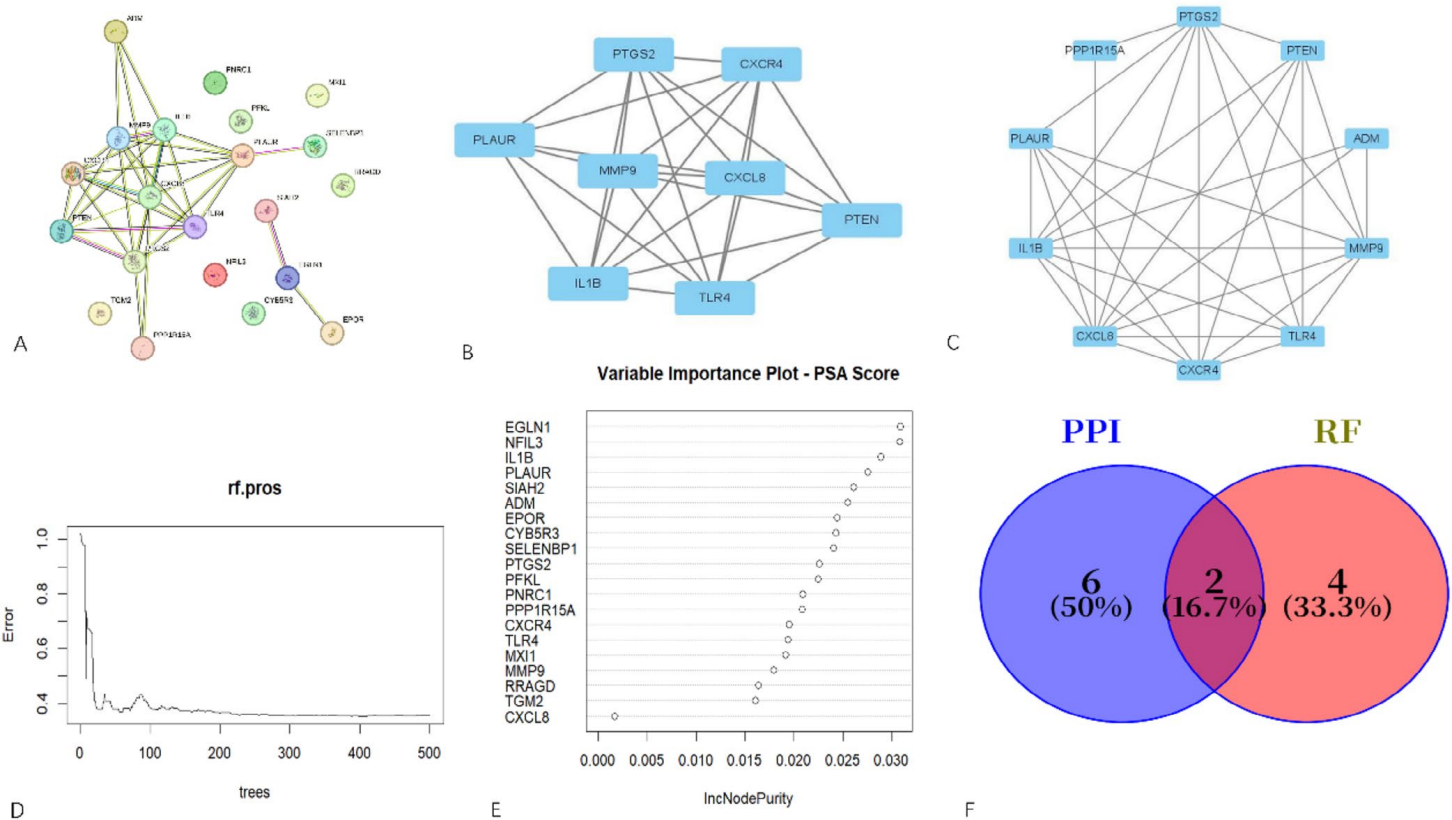
Identification of core hub genes. (A) PPI network visualization. (B) Key gene modules screened by Cytoscape MCODE. (C) Top 10 hub genes via Cytoscape CytoHubba. (D) Tree number‐error rate curve of RF model. (E) Gene importance ranking by RF algorithm. (F) Core hub genes identified via intersection of PPI and RF screened genes.

A PPI network was structured via Cytoscape software based on overlapping DEGs, with the interaction confidence threshold rigorously set at a composite score greater than 0.4. The preliminary network architecture consisted of 14 independent nodes and 35 pairwise interaction relationships (Figure 4A). The embedded MCODE plugin was subsequently utilized to screen clustered functional modules, and one tightly‐connected core module was finally harvested, which contained 8 overlapping DEGs and 27 interactive pairs. The constituent genes of this module were PTGS2, CXCR4, PLAUR, MMP9, CXCL8, PTEN, IL1B, and TLR4 (Figure 4B).

#### 3.3.2 Screening and Profiling of Hub Genes

To screen pivotal hub genes, six computational algorithms integrated in the CytoHubba plugin were adopted to rank genes according to their topological importance, and the top 10 candidate genes were screened out (Table 3). Venn diagram intersection analysis was further conducted between the above candidate hub genes and module genes obtained from MCODE analysis. A total of eight overlapping core genes were identified, namely PTGS2, CXCR4, PLAUR, MMP9, CXCL8, PTEN, IL1B, and TLR4 (Figure 4C).

**TABLE 3:** The top 10 hub genes rank in cytoHubba.

MCC	MNC	Degree	Closeness	Radiality	EPC
CXCL8	CXCL8	CXCL8	CXCL8	CXCL8	MMP9
MMP9	MMP9	MMP9	MMP9	MMP9	CXCL8
IL1B	IL1B	IL1B	IL1B	IL1B	PTGS2
PTGS2	PTGS2	PTGS2	PTGS2	PTGS2	IL1B
CXCR4	CXCR4	CXCR4	CXCR4	CXCR4	CXCR4
TLR4	TLR4	TLR4	TLR4	TLR4	TLR4
PLAUR	PLAUR	PLAUR	PLAUR	PLAUR	PTEN
PTEN	PTEN	PTEN	PTEN	PTEN	PLAUR
ADM	ADM	ADM	ADM	ADM	ADM
PPP1R15A	PPP1R15A	PPP1R15A	PPP1R15A	PPP1R15A	PPP1R15A

#### 3.3.3 Core Gene Screening via Random Forest Algorithm

Feature biomarkers were further screened from the 21 candidate HIRGs using the RF algorithm. Genes with an IncNodePurity value exceeding 0.025 were regarded as functionally significant signatures. In total, six pivotal genes, namely EGLN1, NFIL3, IL1B, PLAUR, SIAH2, and ADM, were successfully identified according to internal node impurity (Figure 4D,E). To narrow down the ultimate hub genes for subsequent validation, overlapping analysis was performed between signature genes derived from the RF model and hub candidates obtained from the PPI network. After intersection matching, PLAUR and IL1B were determined as the two central core genes implicated in AMI pathogenesis (Figure 4F).

### 3.4 Evaluation of Diagnostic Efficacy for Core Genes

**FIGURE 5:**
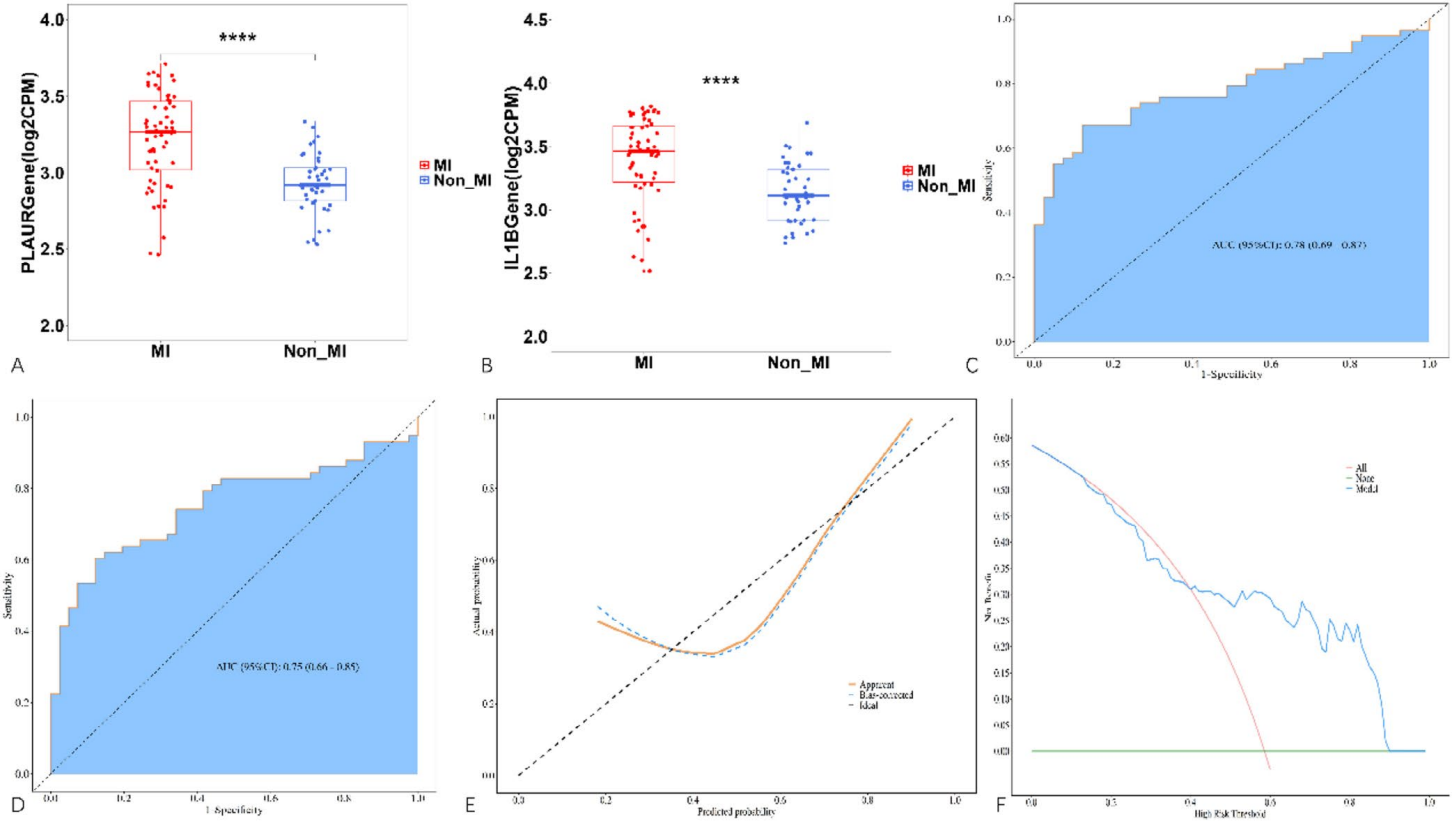
Diagnostic efficacy of PLAUR and IL1B in AMI. (A,B) Upregulated PLAUR and IL1B in GSE66360 validation cohort, ****p < 0.0001. (C,D) ROC curves and AUC values for their diagnostic ability. (E) Calibration curve showing good consistency of the two‐gene model. (F) DCA curves comparing clinical benefits of single and combined models.

Consistent expression trends were observed between the training and external validation cohorts. The two hub genes PLAUR and IL1B exhibited markedly elevated expression levels in specimens from acute myocardial infarction patients, with statistically significant intergroup disparities (Figure 5A,B).

Receiver operating characteristic (ROC) curve analysis was implemented for the two signature genes PLAUR and IL1B, and corresponding area under the curve (AUC) values were quantified (Figure 5C,D). Biomarkers with an AUC exceeding 0.7 alongside a *p‐*value below 0.05 were defined as possessing reliable diagnostic capacity for AMI. Both candidate genes met this standard: PLAUR yielded an AUC of 0.78 (*p* < 0.0001), while IL1B presented an AUC of 0.75 (*p* < 0.0001), verifying their diagnostic potency for acute myocardial infarction.

The calibration curve showed that the apparent and bias‐corrected curves closely matched the ideal diagonal line, demonstrating good predictive calibration of the *PLAUR*‐IL1B model for AMI (Figure 5E). DCA revealed that the *PLAUR*‐IL1B model produced higher net clinical benefit than the “treat all” and “treat none” strategies across all risk thresholds (0–1.0), indicating the reliable clinical application value of the present model (Figure 5F).

### 3.5 Successful Establishment of the Myocardial Infarction Mouse Model

**FIGURE 6:**
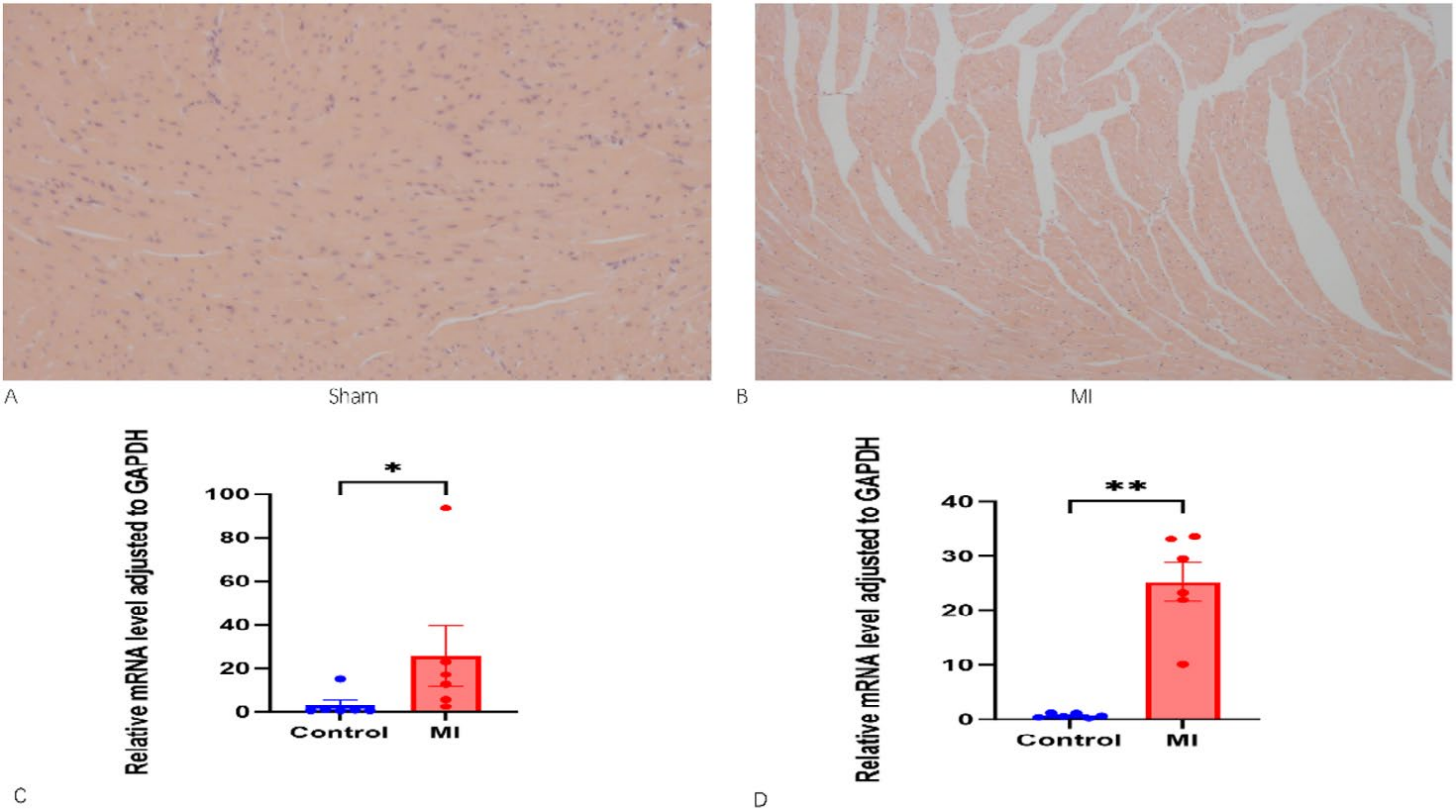
Core gene expression validation. (A) HE staining of sham group myocardium. (B) HE staining of MI group myocardium. (C) qPCR confirmed elevated PLAUR mRNA in MI tissues (*p < 0.05). (D) IL1B was significantly upregulated in MI myocardium (**p < 0.01).

Results of HE staining revealed that myocardial cells in the sham‐operated group maintained normal morphology, size, and structure (Bai et al. 2021) (Figure 6A), whereas those in the myocardial infarction group exhibited extensive necrosis, marked cellular degeneration, and hypertrophy (Figure 6B); disorganized arrangement of myocardial cells and significantly reduced survival rate confirmed the successful establishment of the myocardial infarction mouse model (Gao et al. 2010).

### 3.6 qPCR Validation Results

qPCR analysis was performed on six myocardial infarction group tissue samples and six matched sham surgery group tissue specimens for validating the mRNA expression levels of PLAUR and IL1B. Findings showed that PLAUR and IL1B expression was significantly upregulated in the myocardial infarction cohort (*p* < 0.05), while expression amounts were lower in the sham surgery group (Figure 6C,D).

## 4 Discussion

MI represents a leading global cause of cardiovascular mortality. Hypoxic–ischemic injury and subsequent inflammatory cascades trigger cardiomyocyte necrosis, adverse ventricular remodeling, and progressive heart failure. In this study, two human peripheral blood transcriptomic datasets were integrated to identify 633 AMI‐associated DEGs. After cross‐comparison with a hypoxia‐ischemia gene repository, 21 HIRGs were obtained. Hub genes PLAUR and IL1B were screened via combined PPI network analysis and random forest algorithm. ROC analysis based on an external dataset verified their favorable diagnostic performance. A murine AMI model was established, and qPCR assays confirmed markedly elevated transcription of both genes in ischemic myocardium. Collectively, bioinformatic profiling and in vivo animal experiments demonstrate that PLAUR and IL1B act as critical mediators governing early hypoxic–ischemic inflammatory injury post AMI.

PLAUR (plasminogen activator receptor‐like uric acid receptor), a key molecule in the fibrinolytic system (Dai and Lin 2023), may exacerbate myocardial injury during MI ischemia‐hypoxia through two pathways: On one hand, PLAUR activates plasminogen to generate plasmin, and excessive fibrinolysis disrupts myocardial tissue matrix integrity, leading to infarct expansion (Hindy et al. 2022); on the other hand, PLAUR can also activate the PI3K‐Akt/NF‐κB signaling pathway via non‐fibrinolytic pathways, promoting the secretion of inflammatory mediators (like IL‐6 and TNF‐α) and amplifying the post‐ischemic inflammatory response (Penkov et al. 2024). As a classic proinflammatory cytokine (Tylutka et al. 2024), IL‐1β is directly regulated by HIF‐1α under hypoxic conditions—HIF‐1α expression increases during ischemia‐hypoxia, associating with the hypoxia response component (HRE) in the IL‐1β promoter sequence to promote IL‐1β transcription and secretion (Torretta et al. 2020). Secreted IL‐1β further binds to IL‐1 receptors on cardiomyocyte surfaces, activating the MAPK/JNK signaling pathway. This induces cardiomyocyte apoptosis, inhibits vascular endothelial cell proliferation, impedes neovascularization in the infarct zone, and ultimately exacerbates myocardial ischemia‐hypoxia injury (Wang et al. 2021).

Current routine clinical modalities for AMI diagnosis and risk stratification encompass cardiac troponin measurement, 12‐lead electrocardiography, coronary angiography, and validated scoring systems including GRACE and TIMI (Bergström et al. 2026). These tools mainly rely on anatomical abnormalities, electrographic alterations or markers of extensive cardiomyocyte necrosis, failing to reflect the underlying hypoxic‐inflammatory molecular phenotype of patients. Elevated troponin merely indicates irreversible myocardial injury (Hessel et al. 2008), and cannot predict progressive inflammatory deterioration prior to overt necrosis. In contrast, transcriptional upregulation of PLAUR and IL1B is triggered rapidly after coronary occlusion. As a master upstream pro‐inflammatory cytokine, IL1B recruits neutrophils and amplifies myocardial inflammatory infiltration (Usui et al. 2019), whereas PLAUR modulates plasmin‐driven extracellular matrix degradation, endothelial dysfunction, and inflammatory cell migration within the ischemic microenvironment (Jeon et al. 2023). Quantification of peripheral blood transcript levels of these two genes enables earlier identification of patients prone to severe inflammatory decompensation. During emergency triage, intensified monitoring, personalized anti‐inflammatory regimens and enhanced cardioprotective strategies can be administered to such high‐risk subgroups, a clinical advantage unachievable by conventional necrosis biomarkers.

Translation of transcriptomic biomarkers into routine bedside management necessitates composite predictive models integrating traditional clinical covariates and transcriptional signatures. Machine learning and deep learning architectures have been proven to refine risk stratification for acute cardiovascular disorders and perioperative cardiac injury, establishing a well‐established framework for constructing our AMI signature model. A landmark machine learning‐derived risk calculator was developed to assess perioperative myocardial injury among elderly patients undergoing non‐elective surgery (Cicek et al. 2024); integration of routine clinical parameters and circulating molecular biomarkers markedly improved predictive accuracy compared with conventional scoring systems alone, resolving systematic risk underestimation in vulnerable subgroups. Similarly, deep neural networks greatly boost short‐term mortality prediction for acute pulmonary embolism (APE) (Cicek et al. 2025). APE and AMI are both life‐threatening thrombo‐inflammatory acute vascular conditions, sharing overlapping pathophysiological cascades involving ischemia, thrombosis, and inflammation (Burke and Virmani 2007; Giordano et al. 2017). Drawing upon this analytical paradigm, our team will construct a multivariable predictive model incorporating canonical clinical predictors (age, hypertension, diabetes mellitus, peak troponin concentration, TIMI flow grade) alongside expression values of PLAUR and IL1B. Rigorous internal cross‐validation and large‐scale prospective external cohort validation will be performed to calibrate risk cut‐off values and guarantee stable predictive performance across diverse patient populations and medical centers.

Several inherent limitations of this study should be acknowledged transparently. First, transcriptomic screening was conducted using human peripheral whole‐blood datasets rather than myocardial biopsy specimens from AMI patients. Circulating transcripts reflect systemic inflammatory status rather than exclusive cardiac parenchymal signaling, though our in vivo myocardial qPCR results confirmed local cardiac upregulation of both genes. Second, only mRNA abundance was quantified in murine tissues; protein expression, subcellular localization, and gain−/loss‐of‐function genetic manipulations were not implemented, precluding definitive verification of their causal regulatory roles in hypoxic myocardial injury. Third, the moderate AUC values (PLAUR: 0.78; IL1B: 0.75) demonstrate that standalone transcript testing cannot replace gold‐standard clinical examinations. Their true translational value lies in additive risk stratification when combined with existing diagnostic platforms.

From a therapeutic perspective, elevated IL1B and PLAUR represent actionable targets for targeted intervention. Anti‐IL1B monoclonal antibodies have exhibited cardioprotective effects and alleviated post‐infarction ventricular remodeling in clinical trials for inflammatory heart diseases (Amrute et al. 2024). Urokinase‐type plasminogen activator receptor (uPAR), the protein encoded by PLAUR, also acts as a promising modulator for cardiac reparative cell therapy (Dergilev et al. 2023). The PLAUR/uPAR axis serves as a potential pharmacological target to modulate the regenerative capacity of cardiac progenitor cells, while IL1B is a clinically validated target for suppressing post‐infarction inflammatory remodeling.

## 5 Conclusion

In summary, this study identifies PLAUR and IL1B as central hub genes governing the interconnected hypoxia–ischemia–inflammation cascade in the early phase of AMI. Rather than superseding troponin assays, electrocardiography or coronary angiography, these transcriptional biomarkers introduce an additional molecular phenotype layer to optimize emergency risk triage. By adopting validated machine learning composite modeling frameworks established for perioperative myocardial injury and acute thromboembolic disease, prospective validation of integrated clinical‐molecular predictive tools can translate our bioinformatic and preclinical findings into practical bedside risk management and individualized cardioprotective treatment for AMI patients.

## Author Contributions

Longsheng Zhang collected and analyzed data, conducted animal experiments, and drafted the initial manuscript. Ning Liang provided revisions to the initial draft and reviewed and revised the final version.

## Funding

This work was supported by the Self‐funded Scientific Research Project of Guangxi Zhuang Autonomous Region (Grant No. Z‐A20261139).

## Ethics Statement

Ethical approval and consent for study participation were reported in accordance with the ARRIVE guidelines (https://arriveguidelines.org). Animal studies adhered to the guidelines outlined in the Guide for the Care and Use of Laboratory Animals and received approval from the Animal Ethics Committee of the First Affiliated Hospital of Guangxi Medical University (approval number 2025‐D0343).

## Conflicts of Interest

The authors declare no conflicts of interest.

## Data Availability

All transcriptomic datasets supporting this study are publicly available in the GEO database under accession numbers GSE48060, GSE97320 and GSE66360 (https://www.ncbi.nlm.nih.gov/geo/). Hypoxia‐ischemia‐related gene sets were retrieved from the GeneCards database (https://www.genecards.org).
